# Getting youth PrEPared: adolescent consent laws and implications for the availability of PrEP among youth in countries outside of the United States

**DOI:** 10.1002/jia2.25363

**Published:** 2019-08-01

**Authors:** Tamara Taggart, Keosha T Bond, Tiarney D Ritchwood, Justin C Smith

**Affiliations:** ^1^ Department of Prevention and Community Health George Washington University Washington DC USA; ^2^ Department of Social and Behavioral Sciences Yale School of Public Health New Haven CT USA; ^3^ Department of Public Health New York Medical College Valhalla NY USA; ^4^ Center for Interdisciplinary Research on AIDS Yale School of Public Health New Haven CT USA; ^5^ Department of Family Medicine and Community Health Duke University School of Medicine Durham NC USA; ^6^ Department of Behavioral Sciences and Health Education Emory University Rollins School of Public Health Atlanta GA USA

**Keywords:** PrEP, PrEP uptake, adolescents, HIV prevention, health policy, low‐ and middle‐income countries

## Abstract

**Introduction:**

Youth under the age of 25 are at high risk for HIV infection. While pre‐exposure prophylaxis (PrEP) has the potential to curb new infections within this population, it is unclear how country‐specific laws and policies that govern youth access to sexual and reproductive health (SRH) services impact access to PrEP. The purpose of this review was to analyse laws and policies concerning PrEP implementation and SRH services available to youth in countries with a high HIV incidence. To the best of our knowledge this is the first systematic assessment of country‐level policies that impact the availability of PrEP to adolescent populations.

**Methods:**

We conducted a review of national policies published on or before 12 June 2018 that could impact adolescents’ access to PrEP, SRH services and ability to consent to medical intervention. Countries were included if: (1) there was a high incidence of HIV; (2) they had active PrEP trials or PrEP was available for distribution; (3) information regarding PrEP guidelines were publicly available. We also included a selected number of countries with lower adolescent HIV incidence. Internet and legal database searches were used to identify policies relevant to adolescent PrEP (e.g. age of consent to HIV testing).

**Results and Discussion:**

Fifteen countries were selected for inclusion in this review. Countries varied considerably in their respective laws and policies governing adolescents’ access to PrEP, HIV testing and SRH services. Six countries had specific polices around the provision of PrEP to youth under the age of 18. Five countries required people to be 18 years or older to access HIV testing, and six countries had specific laws addressing adolescent consent for‐ and access to‐ contraceptives.

**Conclusions:**

Adolescents’ access to PrEP without parental consent remains limited or uncertain in many countries where this biomedical intervention is needed. Observational and qualitative studies are needed to determine if and how adolescent consent laws are followed in relation to adolescent PrEP provisions. Intensified efforts to amend laws that limit adolescent access to PrEP and restrict the establishment of national guidelines supporting adolescent PrEP are also needed to address the epidemic in this group.

## 1 Introduction

Globally, young people (defined by the World Health Organization (WHO) as people aged 10 to 24 years) and adolescents (defined as people aged 10 to 19 years) continue to be disproportionately affected by HIV 1. In 2016, 2.1 million adolescents were living with HIV and 260,000 were newly diagnosed with HIV 2. Adolescence is a developmental period marked by an increased risk for HIV infection due to the convergence of several psychological, social and structural factors 3, 4. Young people who are members of key populations like young men who have sex with men (MSM), young women, young people who inject drugs, and youth who engage in transactional sex are particularly vulnerable to HIV and early HIV‐related mortality 5. Because risk behaviours are often highest during adolescence, people who acquire HIV during adolescence have a greater potential of transmitting the virus to others than those who acquire HIV as adults 5, 6.

Most adolescent HIV prevention interventions either focus on changing individual behaviours (e.g. condom use) or on addressing structural drivers (e.g. cash transfers conditioned on safer sex practices) to decrease the incidence of behaviourally acquired adolescent HIV 5, 7, 8. However, individual‐level interventions have had limited success in impacting HIV incidence, and the impact of structural‐level interventions tends to be long‐term and difficult to assess 5. Further complicating adolescent HIV prevention efforts is that adolescents have limited access to HIV prevention services due to restrictive age of consent laws for sexual and reproductive health (SRH) services 5, 9. For example, in some counties, adolescents may be able to access HIV testing, but are prohibited from accessing HIV treatment without parental consent. Young people under the age of 25 years are also more likely than their child and adult peers to die from AIDS‐related complications 10, 11. While there are a number of programs for children and adults aimed at curtailing the spread of HIV, efficacious interventions for adolescents are sorely needed 5.

Oral pre‐exposure prophylaxis (PrEP) for HIV, a tenofovir‐based antiretroviral drug, offers a promising solution as it prevents HIV acquisition when taken as prescribed 12. Following the WHO recommendation that oral PrEP be offered to people at substantial risk of HIV infection as part of combination HIV prevention approaches, several countries expanded their PrEP implementation strategies to include adolescents 13, 14. The WHO recommendations are not‐age specific, but rather exclusively focus on risk level and key populations 14. These recommendations may be difficult for some countries to implement due to restrictive age of consent laws for medical treatment, HIV testing, and other SRH services. As a result, regulatory approvals for PrEP have moved forward at a rapid pace without sufficient consideration of their appropriateness for youth under the age of legal majority 15. Although there is practical advice on how to implement PrEP at the country level, laws restricting adolescents’ ability to independently consent to SRH services may deter adolescents from seeking PrEP and prevent providers from offering confidential SRH services to their adolescent patients 16, 17, 18. While PrEP represents a promising biomedical intervention with great potential to help decrease worldwide HIV incidence among adolescents, its full effectiveness will only be realized if it is accessible 19. To ensure that adolescents have access to PrEP, we must identify and modify policies that contribute to inequitable access to this potentially life‐saving intervention.

### 1.1 Current review

This review explores attributes of prevailing laws and policies that may impact access to adolescent PrEP in a sample of countries with higher adolescent HIV incidence and a select sample of countries with lower adolescent HIV incidence. Laws include age of consent to sexual intercourse, medical treatment, contraceptives and HIV testing. We also include a review of PrEP national guidelines and adolescent PrEP directives. For this purpose, three sources of information have been triangulated including a review of policies, a review of current country‐specific guidelines, and an analysis of country surveillance and programme monitoring data.

## 2 Methods

### 2.1 Inclusion criteria

We used the following inclusion criteria to select countries: (1) the country had a high incidence of HIV; (2) the country had either active PrEP trials or PrEP was available for distribution; and (3) information regarding PrEP guidelines were publicly available. Our search for policies related to adolescents’ access to PrEP focused largely on countries with the highest burden of adolescent HIV and a select sample of Western countries where PrEP is available. Western countries were selected to explore how countries that have a comparatively low incidence of adolescent HIV, but have a well‐resourced healthcare infrastructure, may address PrEP access among adolescents. We used the WHO and the United Nations’ definition of adolescence, which is classified as the period in human growth and development that occurs after childhood and before adulthood from ages 10 to 19 years 20. Considering that the availability of PrEP in many countries remains limited and the uptake among members of key populations has been slow, we used a broad definition of “policy” to enable us to include all national laws and documents that may govern adolescent PrEP provisions including laws on adolescents’ consent to SRH services and independence in obtaining PrEP.

### 2.2 Search strategy

We conducted an Internet search for national policies related to adolescent PrEP use, its availability, and adolescents’ ability to consent to medical treatment in the identified countries using the following key words: [“Pre‐exposure prophylaxis OR PrEP”] AND [“Policy” OR “Strategies” OR “Guidelines”] AND [“Adolescent” OR “Youth” OR “Child” OR “Teen”]. We specifically targeted reports by the WHO, the President's Emergency Plan for AIDS Relief, and PrEPwatch.org, a website that tracks the global availability of PrEP and ongoing medication trials 21. We searched the official websites of the government or agencies responsible for regulating medical interventions (including prevention, testing and/or screening, and treatment) to identify policies related to PrEP and adolescents’ rights to SRH services. We also searched for policies on the following legal and advocacy databases: GlobaLex, Foreign Law Guide and Global Advocacy for HIV Prevention. For each country, we focused on the most recent policies and guidelines available. Our policy search was conducted from inception until 12 June 2018.

### 2.3 Data extraction

Countries were divided equally among the four coders (TT, KTB, TDR and JCS). We extracted information regarding age of majority, national guidelines and policies for adolescent PrEP, age of consent to SRH services (i.e. contraceptives and HIV testing), medical treatment and diagnostic laws, and PrEP availability. These data were documented in tabular form in Microsoft Excel for analyses. The research team met to discuss findings and exchange information, and adjusted search strategies as necessary.

## 3 Results and discussion

We identified 15 countries for this review. The majority of the countries identified are in Eastern and Southern Africa, and South Asia. Table 1 summarizes the age of consent laws for sexual intercourse, medical treatment, SRH services and HIV testing.

**Table 1:** Age of consent laws for sexual intercourse, medical treatment, sexual and reproductive health services, and HIV testing

Country	Age of consent to sexual intercourse	Age of consent to medical treatment	Age of consent to access contraceptives	Age of consent HIV testing
Australia	16‐17 years. Varies by state (Crimes Act and Criminal Code Act)	Unclear. Varies by state, although age of majority is 18 years (Minors (Property and Contracts) Act 1970; Consent to Medical Treatment and Palliative Care Act 1995)	Unclear. Varies by state, although age of majority is 18 years (Minors (Property and Contracts) Act 1970; Consent to Medical and Dental Procedures Act 1985)	No age limit. HIV screening should be discussed with all adolescents who are sexually active or have a history of injection drug use. Parental or guardian involvement in an adolescent's healthcare is often desirable, but is sometimes contraindicated for the safety of the adolescent, and can compromise full disclosure (The National HIV Testing Policy 2017)
Brazil	14 years (Brazilian Criminal Code, Decree Law 2848, Law 12,015)	18 years. Although, the HIV/sexual transmitted infection program provides access to ART at any age (Sistema Único de Saúde – SUS (Brazilian Universal Health System, created by Law 8808/1990))	No age limit. There is no age restriction in the law for males or females to have access to contraceptives. There is no law requiring parental consent for access of contraceptives. The policy of the public health system provides contraceptives to females in childbearing age, every woman of childbearing age (10 to 49 years) has access to contraceptives (Public Health System, Basic Health Units – Brazilian Federal Constitution and by Law 9263)	12 years (Law 8069/1990; Ministry of Health Ordinance 23)
Ethiopia	18 years. Sex with a minor of the opposite sex aged between 13 and 18 years is a crime. (Criminal Code of 2005; Article 629, Ethiopian Penal Code)	Unclear. Age of the majority is 18 years (Article 257(1) of the Revised Family Code; Criminal Code, Article 545(1))	No age limit. Age of the majority is 18 years	15 years (Guidelines for HIV counselling and testing in Ethiopia. Federal Ministry of Health)
France	15 years (Article 227‐25 of the French Penal Code)	12 years (Article L5126‐4 of the French Penal Code)	Under 18 years can access contraceptive services and emergency contraception without parental consent (Article L5134‐1 of the French Public Health Code)	No age limit. As long as a person can give their free and informed consent (Article 2311‐14 of the French Public Health Code; Ministerial Order Article 1)
India	18 years (Indian Penal Code sections 375‐377)	Unclear. Age of majority is 18 years. However, there is evidence that a child above the age of 12 years can give consent to medical treatment if he or she understands the nature and consequences of the treatment (Age of Majority Act; Indian Penal Code section 89)	Unclear	Unclear. A minor can be tested with parental consent. Age of majority is 18 years (Guidelines issued by National AIDS Control Organization and Age of Majority Act)
Indonesia	Men can consent to sex with a woman at 19 years, and women can consent to sex with a man at 15 years. The age of consent to sex for all same‐sex partnerships is 18 years. However, in some provinces, the age of consent to heterosexual sex is after marriage and all same‐sex sexual activity is illegal (Indonesian Penal Code section 4)	Unclear. People under the age of 18 years have the right to healthcare services, and the right to speak and have their opinions heard and receive, seek and impact information pursuant to their intellect and age. However, the age of majority for entering contracts is 21 (Child Protection Law sections 8 and 10)	Unclear. One law states that only married persons can access contraceptives (Law on Population Development and Development of Family; The Health Law (No. 36/2009))	18 years. Anyone under 18 needs the consent of the parent or guardian for HIV testing (Health Ministerial Decree No. 1507/2005 on Guidelines for Voluntary Counselling and Testing for HIV & AIDS)
Kenya	18 years (Sexual Offences Act section 8)	18 years (Age of Majority Act)	No age limit (Reproductive Healthcare Bill section 4)	18 years. Under 18 may independently consent in writing to an HIV test if she is pregnant, married, a parent or is engaged in behaviour which puts him or her at risk of contracting HIV. Children can consent with written consent of a parent or legal guardian of the child (HIV and AIDS Prevention and Control Act)
Mozambique	18 years (women). Sex with a woman under 18 years is rape (Penal Code and Family Law Act)	Unclear	None	16 years (The Constitution of November 16, 1990)
Nigeria	18 years (The Child Rights Act section 31)	18 years (Criminal Code Act and Code of Medical Ethics)	Unclear	18 years (The Federal Ministry of Health's 2010 National Guidelines for HIV and AIDS; HIV and AIDS (Discrimination) Act No 7)
South Africa	16 years (Sexual Offences and Related Matters Act 32)	12 years (The Children's Act No 38 section 129)	12 years (The Children's Act No 38 section 134)	12 years (The Children's Act No 38 sections 130‐133)
Tanzania	18 years (women) unless married, then the age of consent is 15 or more years (Sexual Offences Special Provisions Act)	None stated, but age of majority is 18 years (AIDS Law Brief, Age of Consent for Voluntary Medical Male Circumcision in Tanzania)	None	18 years. People under 18 years who are sexually active, married, or have children can consent to testing. Under 18 years requires written consent from a parent or recognized guardian (Section 15(2) marriage act, Tanzania National AIDS control programme)
Thailand	15 years (Thai Criminal Code Article 279)	Unclear. Age of majority is 20 years of age. However, minors can consent to some procedure (Civil and Commercial Code)	Unclear	18 years. Under 18 years can independently consent to HIV testing if it is determined that they are capable of understanding the testing process
Uganda	18 years (Penal code section 147)	Unclear (The Public Health Act; The Constitution section 34)	None	12 years (National Policy Guidelines on HIV Counselling and Testing section 4.1)
Ukraine	14 to 17 years, but must have completed pubertal development (Criminal Code article 155 and 156)	Unclear (Article 284 of the Civil Code; Minister of Health's Order No. 382)	Unclear (Article 284 of the Civil Code; Minister of Health's Order No. 382)	14 years (Article 284 of the Civil Code; Law of Ukraine on Combating the Spread of Diseases Caused by Virus Human Immunodeficiency (HIV))
United Kingdom (England)	16 years (Sexual Offences Act of 2003)	16 years. People under 16 years can consent, but it is at the doctor's discretion (Children Act; Family Reform Act; “Gillick Competence”)	16 years	16 years. Under 16 years accessing sexual healthcare (which would include HIV testing as part of a sexual health screen) without a parent or guardian should be assessed for competency to consent (Children Act)

ART, antiretroviral HIV.

### 3.1 Age of consent to sexual intercourse

Age of consent to sexual intercourse is 18 years in seven countries (47%) with some countries’ consent laws differing by type of partnership (e.g. heterosexual and same‐sex partnerships) or gender. For example, according to Indonesian law, men can consent to sex with a woman at 19 years and women can consent to sex with a man at 15 years. Two countries (13%) have laws that prohibit sex with anyone under 15 years. Unlike the other countries in this review, age of consent in the Ukraine is a range between 14 and 17 years and is based on pubertal development.

### 3.2 Age of consent to medical treatment

Laws in seven countries (47%) do not specify an age of consent to medical treatment or the age is unclear. The age of consent to medical treatment in most countries is 18 years. In the Ukraine, people 14 years and older could access SRH services; however, to date, these services do not include PrEP. Other countries’ laws include stipulations for people under 18 years to consent to medical treatment. For example, in the United Kingdom (England) people under 16 years can consent for medical treatment, but it is at the provider's discretion to determine whether they understand the treatment and can provide consent. France is the only country to explicitly include PrEP as part of medical treatment, but their law requires parental consent for medical treatment in people under 18 years.

### 3.3 Age of consent to access contraceptives

Although the majority of countries in this review have national healthcare directives for access to contraceptive services, only six countries (40%) have specific laws addressing adolescent consent for‐ and access to‐ contraceptives. For example, Ukrainian law requires legal guardian or parental consent for anyone aged 14 to 18 years to access SRH services, while Indonesian law restricts access to contraceptives to anyone who is not married.

### 3.4 Age of consent for HIV testing

All 15 countries have national guidelines or laws for age of consent for HIV testing. Age limits range from 12 to 18 years. Of the countries with age limits, five countries (42%) require people to be 18 years or older to consent to HIV testing, while four countries (33%) require those under the age of 18 years who are not married, pregnant or a parent themselves to have parental consent for an HIV test.

### 3.5 PrEP national guidelines

Table 2 provides an overview of PrEP national guidelines and adolescent PrEP directives within each country.

**Table 2:** National PrEP guidelines and national adolescent PrEP guidelines by country

Country	PrEP national guidelines	Adolescent PrEP guidelines
Australia	✓	✓ Potential for side effects, benefits and risk of HIV infection are to be considered when deciding on whether to prescribe PrEP to an adolescent
Brazil	✓	None
Ethiopia	✓	None
France	✓	None
India	None	None
Indonesia	None	None
Kenya	✓	✓ For youth at significant risk of HIV infection, no symptoms of HIV, and are able to adhere to PrEP and willing to attend follow‐up evaluations. Registered for people >15 years and >35 kg
Mozambique	None	None
Nigeria	✓	None
South Africa	✓	✓ For members of key populations which includes adolescent girls and young women; registered for people >15 years
Thailand	None	None
Tanzania	✓	None
Uganda	✓	✓ For members of key populations who are unable or unwilling to achieve consistent use of condoms, such as sex workers, fisher folk, long‐distance truck drivers, men who have sex with men (MSM), uniformed forces, and adolescents and young women engaged in transactional sex. Registered for people > 35 kg
Ukraine	None	✓ PrEP may be prescribed to young people without parental consent if the patient is aged 14 years or older
United Kingdom (England)	✓	✓ People as young as 15 years should be offered PrEP if they are a member of an at‐risk group (e.g. young MSM and TGW) and are identified as being at elevated risk of HIV acquisition through condomless anal sex in the previous 3‐6 months, ongoing condomless anal sex, and/or having anal sex with partners who are HIV positive (based on ART and viral load)

✓ indicates yes.

PrEP, pre‐exposure prophylaxis; TGW, transgender women.

Ten countries (66%) have national guidelines for PrEP to assist providers with patient care. Of these, six countries (60%) include specifications for people under the age of 18 years. For example, in the Ukraine, PrEP can be provided to people 14 years and older who are at risk for HIV. In Australia, guidelines do not list a specific age limit for PrEP; rather, guidelines offer considerations for side effects, health benefits and risk of HIV infection when providing PrEP to adolescents. In South Africa and the UK, national guidelines recommend that PrEP be offered to adolescents who are members of key populations (e.g. young MSM and young women).

### 3.6 Discussion

Currently, indications for PrEP including initial and follow‐up prescribing and testing recommendations are the same for adolescents and adults 22. However, adolescents’ access to PrEP is dependent upon several external factors including parental consent and involvement, confidentiality, and access to healthcare providers who are knowledgeable and trained to prescribe PrEP 23, 24. Even in countries where adolescents can independently consent to PrEP, providers may be reluctant to prescribe due to concerns about medication adherence, ability to understand the risks and benefits of PrEP, and risk compensation 25. Additional concerns that potentially limit adolescents’ access to PrEP include stigma, psychological burden and potential adverse health effects such as decreased bone mineral density 13, 22, 26.

Despite these concerns, PrEP represents a promising biomedical intervention for addressing the global adolescent HIV epidemic. With the onset of regulatory approval of adolescent PrEP in the United States, understanding the laws which govern access and adherence to PrEP is a necessary and critical next step 27. To the best of our knowledge, this is the first review of laws and national guidelines on adolescent PrEP in countries with some of the highest rates of adolescent HIV. Our review included a cross‐section of age of consent laws that affect adolescent PrEP delivery services including consent to HIV testing, SRH services and general medical treatment. Study findings indicate a need for: (1) the amendment of age of consent laws that also require parental consent to ensure that safeguards (e.g. providers protecting adolescents’ confidentiality) are delineated 25, (2) the amendment of laws to support prophylaxis, not as medical treatment for HIV, but as prevention of HIV, and (3) consistency in age of consent laws that differentiate between gender and/or sexual orientation and ability to consent.

Most countries in this review have written national guidelines for implementing PrEP. Many of these guidelines include directives for key populations such as at‐risk adolescents. However, age of consent laws requiring parental consent for HIV testing and medical treatment undermine these directives. Adolescents may be unwilling to disclose their sexual activity to their parents because of fear, sexual stigma and discomfort 28. Requiring parental consent may also dissuade adolescents from seeking basic SRH services and raises concerns about physician‐patient confidentiality 21, 29. In fact, recent findings from ATN 113, a clinical trial to explore the safety, acceptability and feasibility of PrEP among adolescents aged 15‐17 years, showed that requirements for parental/guardian consents may inhibit access and uptake of PrEP due to unwanted disclosure of sexual activity and sexual orientation 30. Alternatives to parental consent, such as waivers to consent and provider reporting mandates, especially for adolescents who may be engaging in consensual but underage sexual activity, are needed to ensure adolescent PrEP uptake.

Across laws and national guidelines, classification of PrEP as biomedical HIV prevention rather than HIV treatment is inconsistent. This inconsistency has implications for provider prescribing behaviours due to ethical and legal differences between HIV prevention and treatment options, challenges identifying appropriate PrEP‐eligible patients and cost barriers for youth under 18 years 15, 31, 32. In the United States, for example, all states allow some adolescents to consent to receive testing or treatment for sexual transmitted infections (STI) 21; however, states vary with regard to whether adolescents are able to consent to HIV‐related services, as some states have yet to classify HIV as an STI. Moreover, only eight states allow adolescents to consent to preventive or prophylactic services 21. In countries where adolescent consent is determined by provider discretion, with the exception of the United Kingdom (England) and France, there is no evidence of legal guidance for providers to systematically assess an adolescent's ability to consent or determine parental autonomy 33. Although there has been an increase in professional guidance for providers to assess an adolescent's ability to consent to clinical care and PrEP, it remains unclear if this guidance has led to an increase in adolescent PrEP provisions 26, 34, 35. Nevertheless, new language should be added to current minor consent laws with consideration of the multilevel vulnerabilities (e.g. disclosure of sexual activity to parents or guardians; or report of underage consensual sexual activity as a criminal offense) that adolescents face in accessing SRH services. Additional global guidance is needed to standardize the classification of PrEP as HIV prevention, and to advocate for the amendment of laws governing age of consent to SRH services that do not reflect the healthcare needs of adolescents who are vulnerable to HIV.

Our review identified age of consent laws that differ by an adolescent's age, gender and sexual orientation and laws that criminalize same‐sex sexual activity. Discordant age of consent laws that criminalize consensual sexual activity among adolescents drives HIV risks and are barriers to accessing SRH services, including PrEP 28, 36. Reporting mandates for providers and other sexual health practitioners hinders their ability to provide confidential SRH services to adolescents, creates mistrust and fear in adolescents towards the medical system, and may further impede adolescents from seeking care when they are in criminalized consensual relationships with other adolescents 16, 37, 38. For example, in Mozambique, age of consent for HIV testing is lower than age of consent to sexual activity. Meaning, a younger adolescent may consent to HIV testing, but in doing so will disclose their sexual activity to a provider who will need to report this activity to law enforcement. Furthermore, decriminalizing same‐sex sexual activity is a recognized important step to addressing sexual stigma and other challenges to accessing adolescent PrEP 28. For women, younger age of consent laws for sexual intercourse perpetuate age‐disparate or intergenerational relationships which increase HIV risk 39, 40. Given that much of the adolescent HIV incidence exists within young MSM and young women, differential laws based on sexual orientation and gender limit access to basic HIV prevention services and care. Global efforts to increase support for adolescents seeking PrEP must also be accompanied by efforts to decriminalize same‐sex sexual activity, challenge and seek to change social norms that support age discordant relationships, and increase community‐level interventions that address the root causes of these relationships and norms.

## 4 Conclusions

This study provides a review of age of consent laws and PrEP initiatives based on a comprehensive internet and legal database search; however, we did not review case law or grey literature that were not available in English. This review did not examine sociocultural drivers of policy such as religion or HIV stigma, nor did we examine the structure of various healthcare delivery systems. Analysis of these factors is beyond the scope of this review and should be considered for future research as they may influence laws and guidelines on PrEP. Lastly, this review reports age of consent laws as written. We are not able to determine how these laws are implemented, how closely providers follow these laws, or if they truly impact access to SRH services including PrEP provisions for adolescents. These limitations lead to some uncertainty about adolescent PrEP implementation in light of current age of consent laws.

The degree to which youth have access to PrEP remains unclear in many countries where this biomedical intervention is needed. Given that adolescent HIV is acquired chiefly through sexual transmission, scaling up PrEP among adolescent populations could significantly curb new infections within this population. This review identifies a number of ambiguities in current laws that may influence the provision of PrEP to youth, and a need for further consideration of adolescent‐specific PrEP issues. Adolescents at substantial risk of HIV are excluded from services for multiple reasons including their age, lack of information on SRH services, discriminatory attitudes of service providers, gender inequality and sexual orientation criminalization. Intensified efforts to modify national and sub‐national laws related to adolescent informed consent are needed to ensure that policy constraints associated with adolescent PrEP are minimized. It is imperative that policy makers, policy implementers, providers and advocates meaningfully engage youth in devising strategies to address the structural (e.g. policy and national guidelines) challenges of implementing adolescent PrEP programmes. More support for providers is needed in order to facilitate trustful and empathetic relationships with their adolescent patients 34. These relationships are particularly important in countries where there is a risk of criminalization due to sexual orientation or where providers are tasked with assessing an adolescent's decision‐making capacity for care 34. Adolescent PrEP providers should ensure confidentiality and continually seek feedback from their adolescent patients on how services and provider‐patient interactions might be improved. PrEP services for youth should endeavour to be youth‐friendly, supportive and include services such as tailored adherence support and flexible visit schedules. Future research is needed to better determine whether: adolescent age of consent laws for sexual intercourse, SRH services, and medical treatment truly influence clinical practice; these laws are associated with the establishment and implementation of PrEP national guidelines; and if these laws influence the provision of PrEP to PrEP‐eligible adolescents.

## Competing interests

The authors have no conflicts to disclose.

## Authors’ contributions

TT conceived and designed the study, conducted data collection and analysis, wrote sections of the paper, and reviewed and approved the final draft of the manuscript. KTB conducted data collection, drafted the introduction and implication sections, and reviewed and approved the final draft of the manuscript. TR conducted data collection, drafted the methods section, edited the manuscript and reviewed and approved the final draft of the manuscript. JCS conducted data collection, edited the manuscript, and reviewed and approved the final draft of the manuscript.
